# Gait Biomechanics and Patient-Reported Function as Predictors of Response to a Hip Strengthening Exercise Intervention in Patients with Knee Osteoarthritis

**DOI:** 10.1371/journal.pone.0139923

**Published:** 2015-10-07

**Authors:** Dylan Kobsar, Sean T. Osis, Blayne A. Hettinga, Reed Ferber

**Affiliations:** 1 Faculty of Kinesiology, University of Calgary, Calgary, Alberta, Canada; 2 Faculty of Nursing, University of Calgary, Calgary, Alberta, Canada; 3 Running Injury Clinic, Calgary, Alberta, Canada; Universite de Nantes, FRANCE

## Abstract

**Objective:**

Muscle strengthening exercises have been shown to improve pain and function in adults with mild-to-moderate knee osteoarthritis, but individual response rates can vary greatly. Predicting individuals who respond and those who do not is important in developing a more efficient and effective model of care for knee osteoarthritis (OA). Therefore, the purpose of this study was to use pre-intervention gait kinematics and patient-reported outcome measures to predict post-intervention response to a 6-week hip strengthening exercise intervention in patients with mild-to-moderate knee OA.

**Methods:**

Thirty-nine patients with mild-to-moderate knee osteoarthritis completed a 6-week hip-strengthening program and were subgrouped as Non-Responders, Low-Responders, or High-Responders following the intervention based on their change in Knee injury Osteoarthritis Outcome Score (KOOS). Predictors of responder subgroups were retrospectively determined from baseline patient-reported outcome measures and kinematic gait parameters in a discriminant analysis of principal components. A 3–4 year follow-up on 16 of the patients with knee OA was also done to examine long-term changes in these parameters.

**Results:**

A unique combination of patient-reported outcome measures and kinematic factors was able to successfully subgroup patients with knee osteoarthritis with a cross-validated classification accuracy of 85.4%. Lower patient-reported function in daily living (ADL) scores and hip frontal plane kinematics during the loading response were most important in classifying High-Responders from other sub-groups, while a combination of hip, knee, ankle kinematics were used to classify Non-Responders from Low-Responders.

**Conclusion:**

Patient-reported outcome measures and objective biomechanical gait data can be an effective method of predicting individual treatment success to an exercise intervention. Measuring gait kinematics, along with patient-reported outcome measures in a clinical setting can be useful in helping make evidence-based decisions regarding optimal treatment for patients with knee OA.

## Introduction

Muscle strengthening exercises have become an integral part of the management of mild-to-moderate knee osteoarthritis (OA) [1]. Previous research studies have reported that patients with knee OA commonly demonstrate improvements in patient-reported pain and physical function following a muscle strengthening exercise intervention [1–5]. However, the effect sizes in many of these trials remain small, most likely the result of the large variability in the individual responses rather than a limitation of the intervention itself [6]. This variability in treatment success has led to a recent paradigm shift from a “one size fits all” model to a personalized medicine approach, with a focus on identifying responders or non-responders [6–8]. While some research has suggested patient-reported outcome measures (e.g., pain ≥ 6/10) may be effective in predicting responders to exercise in hip OA populations [9], this relationship remains unclear [10]. Therefore, further research is needed to validate patient-reported outcome measures and to identify additional variables that may help in the developmental phase of clinical prediction rules for a patient with knee OA’s response to exercise [11].

It is well accepted that patients with knee OA have altered gait, but much like the responses to exercise there is considerable heterogeneity across previous research investigations. While differences in sagittal and frontal plane kinematics have been observed between those with and without knee OA [12–18], as well as between OA severities [12,15–17], compartments [13,18], and bilateral vs. unilateral OA [14,19], a recent systematic review and meta-analysis demonstrated a lack of consistency across studies, suggesting patients with knee OA exhibit a wide variety of biomechanical changes in response to the disease [20]. Similarly, Sagawa Jr. et al. [21] reported that patients with knee OA can adopt a variety of unique gait profiles in the sagittal and frontal plane. Moreover, examining inter-subject variability in kinematic waveforms (e.g., principal component analysis; PCA) has been shown to be useful in discriminating [22] and subgrouping knee OA gait patterns [23]. Gaudreault et al. [23] reported that by grouping patients with similar gait patterns at baseline, significant changes in gait were evident post-intervention that could not have been observed otherwise. Therefore, differences in biomechanical gait profiles observed at the beginning of an exercise intervention may be important predictors of response to the intervention, and complement patient-reported outcome measures.

Hip muscle strengthening is one type of exercise intervention that has been shown to be effective in reducing the pain of patients with knee OA, but there remains no information on identifying individuals who respond best [24,25]. Bennell et al., [24] found significant improvements in pain and function overall following a hip strengthening exercise intervention, but they did not examine differences in subjects at baseline and acknowledged that such differences may have been informative of those patients that responded best. The authors suggested that future work should examine baseline gait parameters as a method to predict potential responders to a hip strengthening program. To the our knowledge, no study has yet to examine whether patient-reported outcome measures and walking gait kinematic data for patients with knee OA are predictive of response to a hip strengthening exercise intervention.

Therefore, the primary purpose of this study was to use pre-intervention sagittal and frontal plane kinematics, in addition to patient-reported outcome measures, to predict post-intervention response to a 6-week hip strengthening exercise intervention in patients with mild-to-moderate knee OA. It was hypothesized that a unique combination of these parameters obtained at baseline would define a subspace that would successfully classify patients with knee OA as Non-Responders, Low-Responders, or High-Responders to the exercise intervention. The secondary purpose of the study was to examine the stability of the knee OA group centroids in the classification subspace following the 6-week intervention, as well as at a 3–4 year follow-up in a limited subset.

## Methods

### Ethics Statement

The experimental protocol was approved by the Conjoint Health Research Ethics Board at the University of Calgary and written informed consent was obtained from all participants prior to testing. The individual demonstrating the exercise protocol in S1 File has given written informed consent to publish these images.

### Subjects and Design

**Fig 1:**
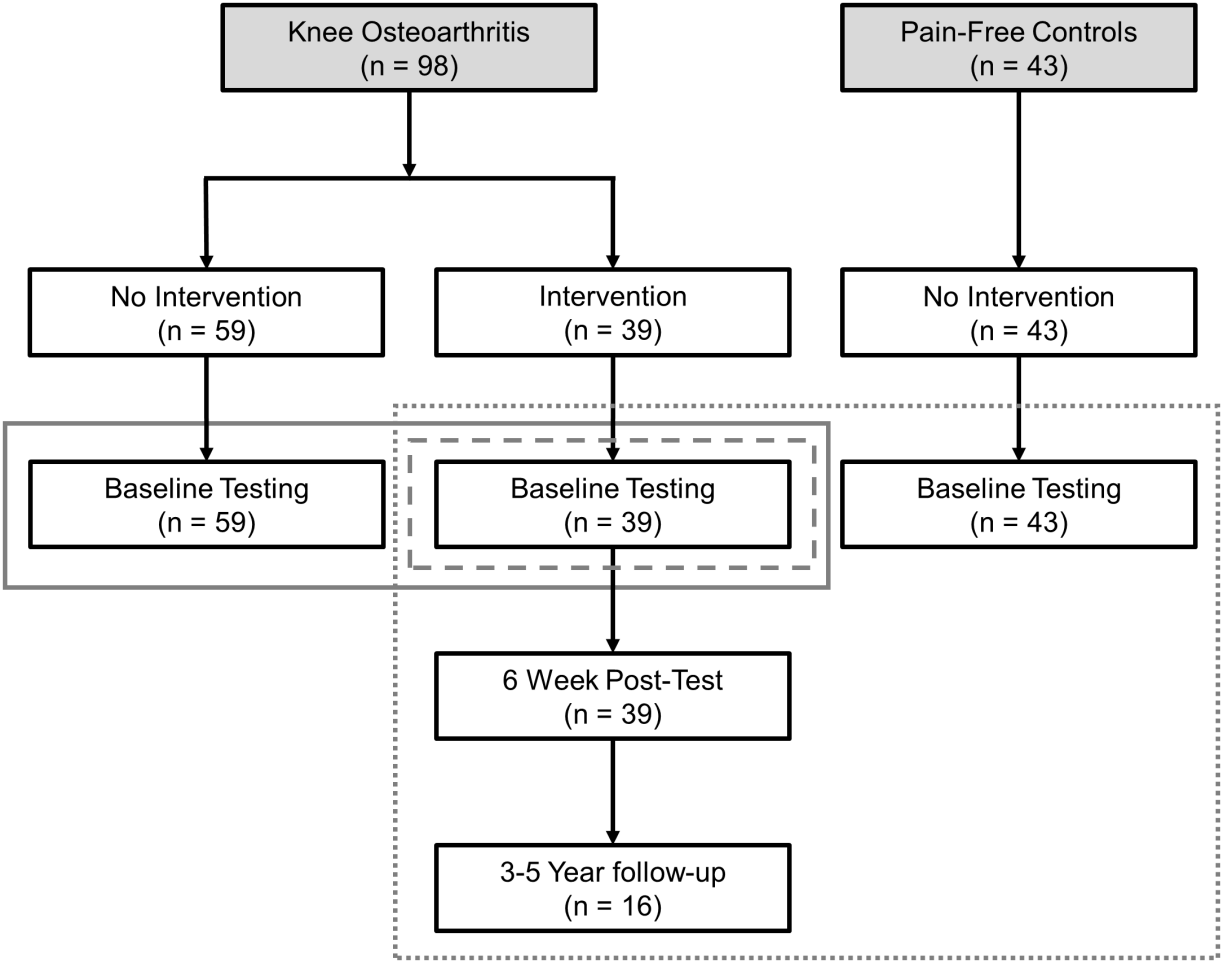
Flowchart illustrating the testing of patients with knee OA and pain-free controls. The three outlining boxes depict use of participants for data reduction (solid), classification algorithm (dashed), and projection in classification subspace (dotted).

A total of 98 adults with knee OA (53 females and 45 males) who were ≥ 40 years of age and met the American College of Rheumatology clinical criteria for mild-to-moderate knee OA in at least one knee [26] were recruited from a database held at an orthopaedic knee clinic at the University of Calgary. Thirty-nine of the patients with knee OA completed a 6-week hip strengthening exercise intervention. The remaining 59 patients with knee OA were not enrolled in the exercise intervention and completed a single baseline testing to be used in the data reduction step to be described in the Data Analysis section (Fig 1). All participants were required to be able to walk without assistive devices and have no physical or medical condition for which the testing protocol would be contraindicated. Knee OA participants were excluded if they (1) were diagnosed with severe knee OA, (2) were currently undertaking physiotherapy or other conservative management practices, including corticosteroid injections, (3) had taken oral corticosteroids or anti-inflammatories in the 24 hours prior to testing, (4) had systemic arthritic conditions, or (5) had a body mass index (BMI) > 35 kg/m^2^. Forty-three pain-free controls (25 females, 18 males) were also recruited from the aforementioned database to be used as reference data to compare to the patients with knee OA (Fig 1). These pain-free controls were ≥ 40 years of age with no knee pain or prior history of knee injury/surgery and no known OA of any lower extremity joint or spine. Univariate comparisons of demographic information between knee OA patients and pain-free controls (independent t-tests), as well as between responder subgroups and pain-free controls (one-way analysis of variances) can be found in Table 1.

**Table 1:** Means (SD) of demographics for all patients with knee OA (n = 98) and pain-free controls (n = 43), as well as responder subgroups in the subset of patients with knee OA who were enrolled in the exercise intervention (n = 39).

Groups (n)		Age (yrs)	Speed (m/s)	BMI (kg/m^2^)	Sex (% F)	OA Compartment (% of patients with Med-Lat-PF-Multi)
Controls (43)		53 (10)	1.16 (0.04)	24.8 (3.7)	58%	
OA (98)		56 (7)	1.13 (0.07)*	26.9 (3.6)*	54%	
	Non-Responders (14)	58 (7)	1.16 (0.03)	25.5 (2.2)	57%	43–7–29–21
Intervention OA (39)	Low-Responders (14)	53 (7)	1.15 (0.03)	24.5 (1.7)	57%	50–0–14–36
	High-Responders (11)	57 (6)	1.12 (0.07)	27.5 (3.5)	100%	64–0–0–36

*OA significantly different from Controls (p < 0.05).

Abbreviations: Med = Medial Compartment; Lat = Lateral Compartment; PF = Patellafemoral Compartment; Multi = Multicompartmental.

### Procedure

All patients with knee OA (n = 98) and pain-free controls (n = 43) had three-dimensional lower limb kinematics collected at 120 Hz using an 8-camera Vicon MX3 (Vicon Motion Systems, Oxford, UK) motion capture system for 60 seconds while walking on an instrumented treadmill (Bertec, Columbus OH, USA). Participants walked at a self-selected normal speed chosen during a familiarization period prior to recording. A total of 27, 9 mm retro-reflective markers were attached to the pelvis, thighs, shanks, and feet, with an additional 18 markers also attached to anatomical landmarks for a neutral standing trial to identify joint centre locations [27,28]. Marker trajectories were filtered with a 10 Hz low-pass 2nd order recursive Butterworth filter and lower body joint angles were calculated using a rigid-body SVD approach [29], and a Joint Coordinate System [30].

In addition to gait kinematics, patients with knee OA enrolled in the exercise intervention (n = 39) also completed a Knee Injury and Osteoarthritis Outcome Score (KOOS). The KOOS is a 42-item self-administered knee-specific standardized questionnaire (5 Likert boxes) which has been shown to display adequate to high test-retest reliability in a knee OA population [31]. The questionnaire assesses five subscales consisting of pain, symptoms, function in daily living (ADL), sport and recreation function, and knee related quality of life (QoL), where a normalized score is calculated for each subscale (100 indicating no symptoms and 0 indicating extreme symptoms) [32]. In the current study, only four subscales were examined (pain, symptoms, ADL, and QoL) as many of the sport and recreation function questions did not apply to the patients. The 39 patients with knee OA involved in the exercise protocol completed the KOOS prior-to and following the exercise intervention.

### Intervention

Following baseline testing, the 39 patients with knee OA in the intervention protocol began a 6-week therapist-directed hip strengthening exercise program. Participants completed dynamic resistance strengthening exercises for the hip using Thera-Band (The Hygenic Corporation, Akron OH, USA) elastic bands. The exercise protocol focused on the hip abductors, hip flexors, hip external rotators, gluteus medius, and core stability (S1 File). The individual in this manuscript has given written informed consent (as outlined in PLOS consent form) to publish these case details. The exercises were shown to the participants by a Board Certified Athletic Therapist who also oversaw one exercise session per week to monitor pain, compliance, and technique. Progression of exercises, including increases or decreases in sets and repetitions or duration of exercises, and changes in TheraBand (Hygenic Corp, Akron, OH) resistance were at the discretion of the Athletic Therapist, based on patient feedback and symptoms during rehabilitation progression. Participants were asked to perform the exercises daily and record the days they completed the exercises. The average number of days per week the exercises were completed was 5.5 (±1.5). The strengthening program was based on a protocol that has been shown to be effective for improving symptoms in patients suffering from patellofemoral pain syndrome [33]. Similar hip strengthening programs have also been shown to improve pain and function in patients diagnosed with knee OA [24,25].

### Subgrouping

**Fig 2:**
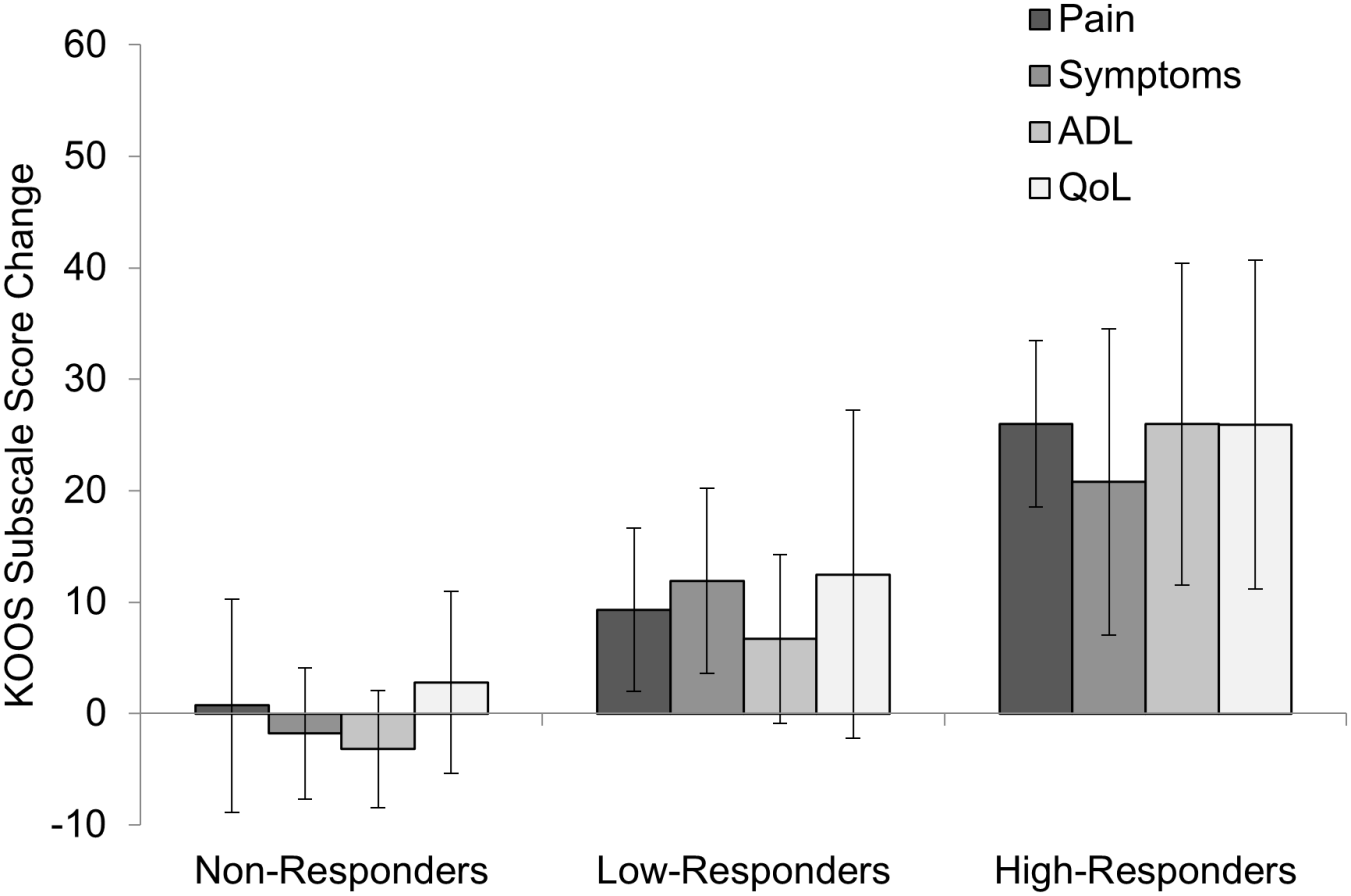
Absolute change in KOOS subscale scores following 6-week intervention, organized by subgroups. Abbreviations: ADL = Function in Daily Living; QoL = Knee Related Quality of Life. Note: KOOS is measured on a scale of 100, with 100 being best (e.g., no pain) and 0 being worst (e.g., most pain imaginable).

Patients with knee OA in the intervention were subgrouped based on their change in patient-reported KOOS subscale scores (pain, symptoms, ADL, and QoL) at the end of the 6-week exercise intervention, as compared to baseline scores [34]. The effect size (Cohen’s d) of the intervention for each participant was averaged across all four KOOS subscales to label participants as Non-Responders (effect size < 0.2), Low-Responders (0.2 ≤ effect size < 0.8), or High-Responders (effect size ≥ 0.8) [35]. This method of subgrouping was applied instead of the OMERACT/OARSI criteria [36] to allow for three potential response groups, as opposed to the binary responder criterion. All but one of the High-Responders met the OMERACT/OARSI criteria to be defined as responders, while no Low- or Non-Responders met these criteria. The absolute change in KOOS subscales following the 6-week exercise intervention for each responder subgroup are shown in Fig 2.

### Data Analysis

**Fig 3:**
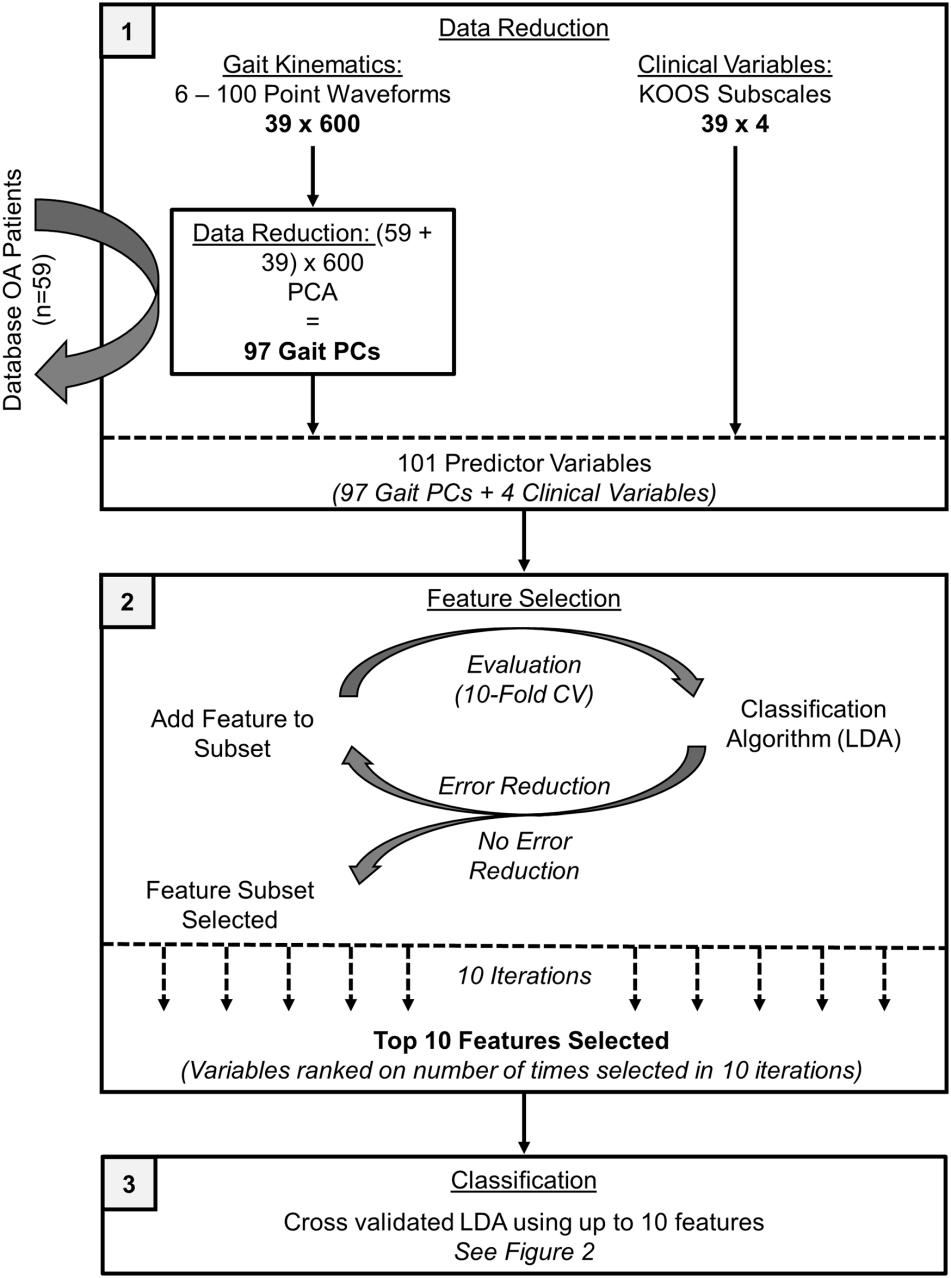
Three primary steps involved in data analysis for the classification of responder subgroups; data reduction, feature selection, and classification.

All data processing was performed using MATLAB 8.4 (The MathWorks Inc., Natick, MA) with statistical analyses performed in IBM SPSS Statistics 21 (SPSS Inc., Armonk NY, USA). The primary purpose of the study, to classify knee OA subgroups as Non-Responders, Low-Responders, or High-Responders using pre-intervention patient-reported outcome measures and gait, was completed in three steps; (1) data reduction, (2) feature selection, and (3) classification as shown in Fig 3.

First, given the large amount of gait data, a principal component analysis (PCA) was used as a data reduction technique [37,38]. A PCA summarizes the variability among participants for a given dataset by creating a new set of orthogonal variables which not only describe, but are ordered based on the variance each explains in the original dataset [37,39]. While studies have successfully used such data reduction techniques with limited sample sizes [40,41], a minimum of 50–100 subjects has been recommend even for highly correlated data [42,43]. Therefore, to limit the risk of overfitting and make the data reduction more generalizable, the gait kinematics of the 59 patients with knee OA who were not involved in the exercise intervention, were included in the PCA.

Prior to conducting the PCA, sagittal and frontal plane hip, knee, and ankle joint angle waveforms from the most affected leg were normalized to stance (60%) and swing (40%) phases and averaged across 10 consecutive strides. The six kinematic waveforms were then combined into a 98 x 600 matrix (patients with knee OA x waveform data points). The matrix was standardized to a mean of 0 and a standard deviation of 1 [44], and a PCA was performed resulting in 97 principal components (PCs). Therefore, the 600 point gait kinematic waveform of each individual at the baseline of the exercise intervention was reduced to 97 PC scores that related to the direction of the largest total variance in knee OA gait and indicated how the individual deviated from the average. These gait PCs were then combined with the four KOOS subscales to create 101 potential predictor variables.

Second, feature selection was accomplished by selecting a subset of predictor variables using a sequential forward feature selection. This technique utilized the same classification algorithm to be used in the final step of data analysis (linear discriminant analysis; LDA), but begins with no predictor variables in the feature subset and sequentially adds a variable that produced the greatest error reduction until adding any additional variable does not reduce this error [45]. The measure of error used in the current study was a 10-fold cross-validation error which was repeated 10 times (i.e., 10x10-fold cross-validation [46]). The use of a cross-validated error measure is critical when selecting features to minimize the statistical bias or overfitting of the final model [47]. All selected features were ordered by percentage of times selected in the 10 iterations and only the top 10 features were selected to meet the minimum requirements of the classification model (i.e., one less than the smallest subgroup size) [48].

Finally, the 10 features selected were used in a classification algorithm to determine the cross-validated classification accuracy of the best subset of features. Specifically, a LDA was used to classify knee OA participants as Non-Responders, Low-Responders, or High-Responders with up to 10 of the selected features. The goal of the LDA was to linearly combine multiple independent variables into k–1 composite variables, called discriminant functions (where k equals the number of responder groups), that best discriminate group membership [49]. All features were normally distributed (Kolmogorov-Smirnov and Lilliefors correction test) with no outliers, both critical assumptions of the LDA [48]. Cumulatively entering features into the LDA, in the order of percentage of times selected in the feature selection step, and selecting the combination with the greatest 10x10 fold classification accuracy determined the best classification model. The significance of the discriminant functions was defined using Wilks’ Lamba (p < 0.05) and equivalence of variance-co-variance matrices was tested using Box’s M statistic (p > 0.05).

The secondary purpose, to examine the stability of the knee OA group centroids, was completed by projecting post-intervention and 3–4 year follow-up data into the best classification model’s subspace. The 39 patients with knee OA enrolled in the exercise intervention were not followed or tracked in any way after their 6-week post-test until being contacted for a onetime testing session (i.e., gait kinematics and KOOS questionnaire) an average of 3.5 (±0.3) years following their involvement in the study. The subset of follow-up patients that was recruited (n = 16) displayed similar BMI and gait speed compared to their baseline testing (Table 2). Pain-free controls were also projected into the classification subspace to increase the clinical interpretation of the model. Before these data could be projecting into the subspace, kinematic data were organized and processed in a manner identical to pre-intervention knee OA data and PC scores were computed from the coefficients of the PCs used in the best classifying LDA. These PC scores depicted how the new kinematic data (i.e., post-intervention, 3–4 year follow-up, pain-free controls) deviated from the average baseline kinematic data (i.e., 98 patients with knee OA) used in conducting the PCA. In addition to gait data, the necessary patient-reported outcome measures (KOOS subscales) were used to compute discriminant functions scores and plot these results in the classification subspace. Since control participants were pain-free, they were assigned perfect scores on the KOOS subscales. Although it is unlikely that all control subjects would reach perfect scores on all KOOS subscales [50], the primary use of this data was as a pain-free control reference for comparing to responder subgroups in the classification subspace.

**Table 2:** Means (SD) of demographics for 3–4 follow-up patients (n = 16) at baseline at 3.5 (±0.3) years following their involvement in the study.

Responder Subgroups (n)		Age (yrs)	Speed (m/s)	BMI (kg/m^2^)	Sex (% F)
Non-Responders (7)	Baseline	58 (7)	1.15 (0.01)	25.7 (2.3)	43%
	Follow-up	62 (6)	1.13 (0.02)	26.0 (3.2)	43%
Low-Responders (7)	Baseline	52 (8)	1.14 (0.03)	24.4 (1.9)	57%
	Follow-up	56 (8)	1.14 (0.03)	23.9 (1.7)	57%
High-Responders (2)	Baseline	61 (6)	1.15 (0.01)	30.6 (5.1)	100%
	Follow-up	65 (5)	1.15 (0.02)	29.0 (6.3)	100%

## Results

**Fig 4:**
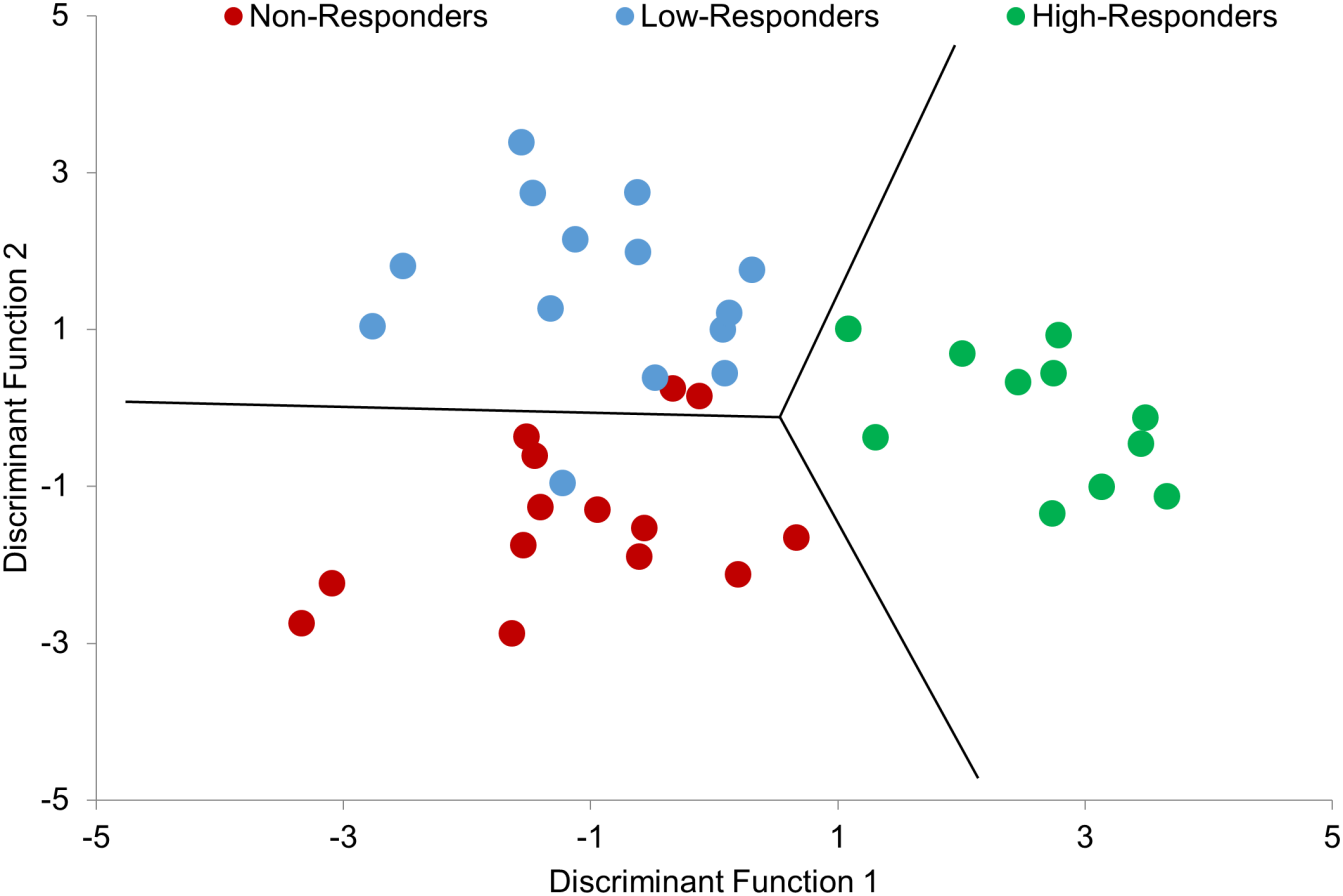
Scatterplot of discriminant function scores computed from pre-intervention patient-reported outcome measure (ADL) and gait of knee OA responder subgroups.

On average, all patients with knee OA walked slower (t (139) = -2.641, p = 0.009) and had a greater BMI (t (139) = 3.066, p = 0.002) than pain-free controls, but no individual responder subgroups differed in age (F (3,78) = 1.563, p = 0.205), gait speed (F (3,78) = 2.314, p = 0.082), BMI (F (3,78) = 2.364, p = 0.078), and sex (F (3,78) = 2.555, p = 0.061; Table 1). The best classifying LDA revealed two significant discriminant functions (Λ = 0.095, χ2 (14, N = 39) = 77.5, p < 0.001), which used 1 patient-reported outcome measure and 6 gait PCs to explain 74.6% of the variation between subgroups and achieve a 10x10 fold cross-validation classification accuracy of 85.4%. Box’s M statistic indicated that the assumption of equivalence of variance-co-variance matrices was not violated (p = 0.39). Discriminant function scores were used to plot patients with knee OA in the LDA classification subspace shown in Fig 4.

**Fig 5:**
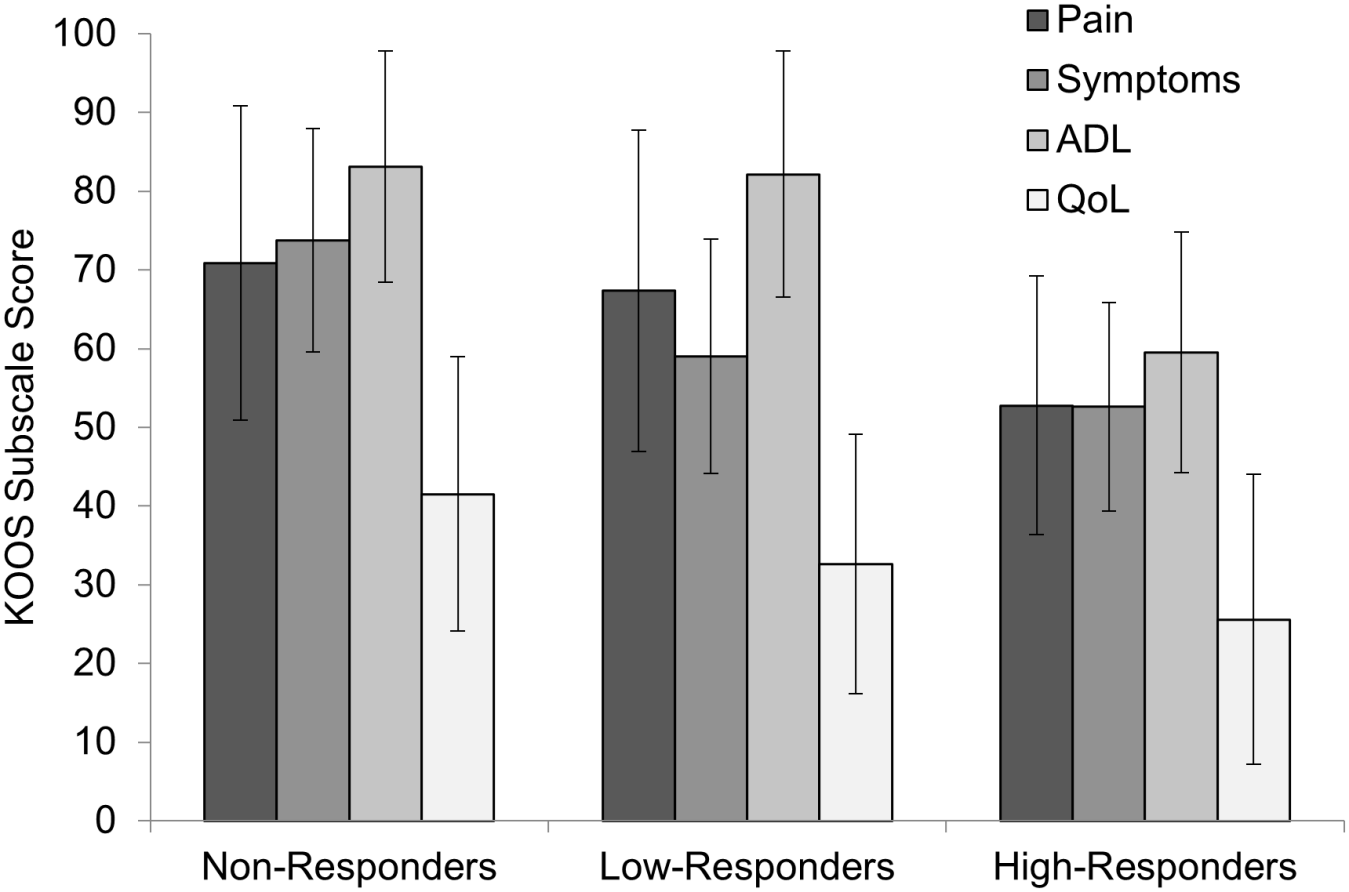
Pre-intervention Knee Injury and Osteoarthritis Outcome Score subscales of pain, symptoms, function in daily living (ADL), and knee related quality of life (QoL) of Non-Responders, Low-Responders, and High-Responders. Note: KOOS is measured on a scale of 100, with 100 best (e.g., no pain) and 0 being worst (e.g., most pain imaginable).

The first discriminant function was primarily related to the KOOS ADL subscale and gait PC 51 (Table 3) and was most important for discriminating High-Responders from other subgroups, as High-Responders displayed the highest scores on discriminant function 1 (Fig 4). Based on the relationship between these variables and discriminant function 1 shown in Table 3, higher discriminant function 1 scores were related to lower (i.e., worse) ADL scores (Fig 5) and higher gait PC 51 scores. Gait PC 51 was associated with the pattern of motion in hip frontal plane kinematics during loading response. High-Responders scored higher on gait PC 51 (p = 0.001) than other subgroups, which related to High-Responders being more likely to display an increasing pattern of hip adduction during loading response, while other subgroups were more likely to maintain or decrease hip adduction during this time.

**Table 3:** Structure matrix of correlations between predictor variables and discriminant functions.

Principal Components	Discriminant Function 1	Discriminant Function 2
ADL	-0.42^a^	0.01
PC51	0.34^a^	0.06
PC11	0.28	0.15
PC22	0.25	0.07
PC46	0.00	-0.47^a^
PC90	0.03	0.40^a^
PC86	0.06	0.30^a^

Abbreviations: ADL = Function in daily living subscale; PC = Gait Principal Component.

^a^ Correlation ≥ 0.3 signifies most important variables on each discriminant function.

**Fig 6:**
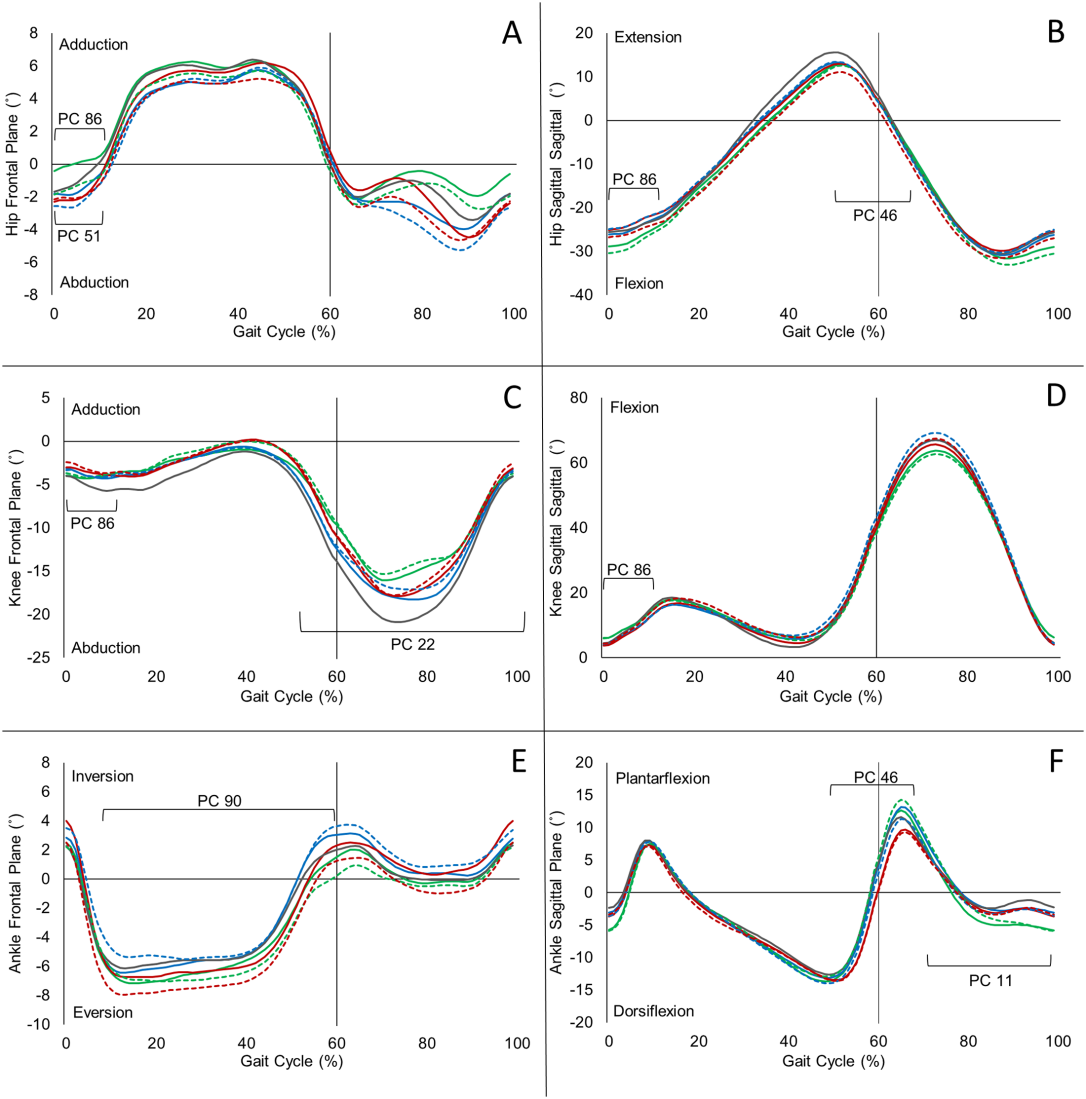
Frontal and sagittal kinematic waveforms for the hip, knee, and ankle of High-Responders (green), Low-Responders (blue), Non-Responders (red), and controls (grey) both pre (solid) and post-intervention (dashed), with peak loadings of predictor principal components illustrated with brackets.

The second discriminant function related to the combination of gait PCs 46, 86, and 90 (Table 3) and was most important for discriminating Non-Responders from Low-Responders, as Low-Responders displayed higher scores on discriminant function 2 than Non-Responders (Fig 4). Gait PC 46 was generally related to a relationship between hip and ankle kinematics at toe-off. Non-Responders scored higher on gait PC 46 (p = 0.003) than Low-Responders suggesting they are more likely to have reduced plantarflexion, in relation to hip extension, at toe-off. The remaining higher-order gait PCs were more difficult to interpret but were primarily loaded on hip and knee kinematics during loading response (gait PC 86) and frontal plane ankle kinematics during stance (gait PC 90). Fig 6 visually illustrates where these gait PCs are most highly loaded on the kinematic waveforms.

**Fig 7:**
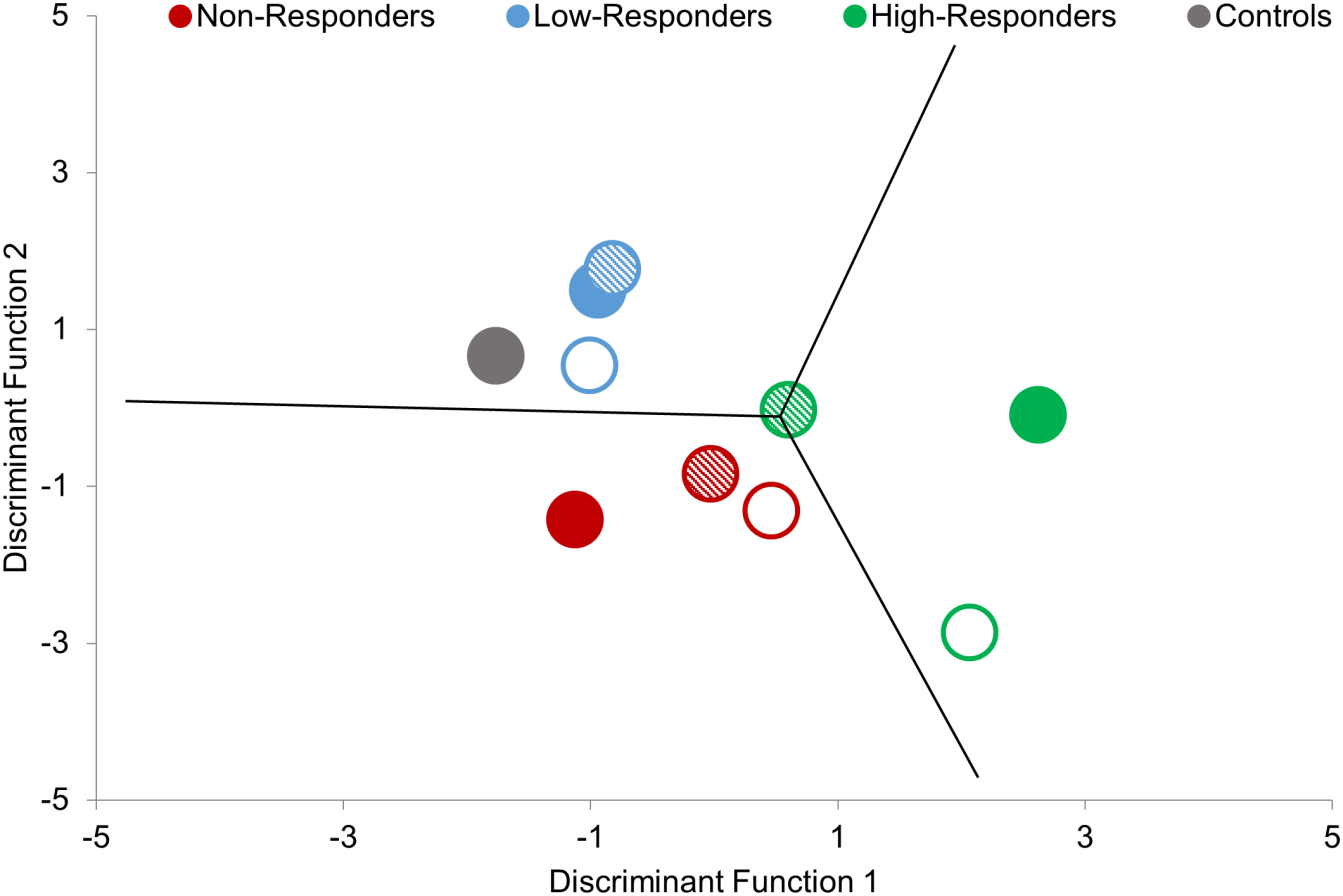
Scatterplot of group centroids for pre-intervention (solid), post-intervention (striped), and 3–4 year recall (open) of knee OA subgroups.

Pain-free control data were projected into the classification subspace in Fig 7. The control group’s centroid was most similar to the Low-Responders, followed by Non-Responders, and lastly High-Responders. Projecting post-intervention knee OA subgroups into the classification subspace demonstrated that although subgroup centroids shifted, they all remained in their initial third of the subspace. Similarly, projecting the subset of patients with knee OA that completed the 3–4 year follow-up (n = 16), revealed all subgroup centroids remained in their initial third of the subspace even after an average of 3.5 (± 0.3) years.

## Discussion

The purpose of this study was to use pre-intervention kinematic gait data and patient-reported outcome measures to classify patients with mild-to-moderate knee OA as Non-Responders, Low-Responders, and High-Responders to a 6-week hip strengthening exercise protocol. In support of our hypothesis, by using a combination of these patient-reported outcome measures and biomechanical parameters we were able to successfully classify (85.4% cross-validated accuracy) responder subgroups. To our knowledge, this is the first study to use patient-reported outcome measures and objective biomechanical gait waveform data to successfully predict individual treatment success to an exercise intervention in knee OA.

In order to create a more efficient and effective model of care for knee OA, it is critical to provide the right patients the right treatment at the right time. Using baseline patient data to classify responders can assist clinicians in directing patients to the most effective treatment, potentially saving time and concomitantly reducing health care costs. The model developed in the current study shows that the use of patient-reported outcome measures and gait data was able to successfully predict responders to a 6-week exercise intervention with greater than 85% accuracy. While we are not aware of previous research studies combining patient-reported outcome measures and gait data, Weigl et al. [51] reported 74.8% classification accuracy for hip and knee OA responders using predictor variables such as sex, depression symptoms, and medical history. Similarly, a study on patients with hip OA by Wright et al. [9] used predictor variables such as age, pain, duration of symptoms, and gait speed to achieve 65% classification accuracy for determining responders. Thus, the results of the current study suggest that patient-reported outcome measures, combined with objective gait data within a relatively heterogeneous knee OA population, may provide a robust method for identifying subgroups of responders and non-responders. Moreover, the current study improves upon the aforementioned investigations [9,51], in that the model provides a three-group solution, rather than two, and the cross-validated classification accuracy uses partitioned subsets of subjects to predict the left-out subset of subjects, thereby limiting overfitting of the model. Nevertheless, this remains a preliminary study depicting cross-validated error and requires test-set validation using a new cohort of patients with knee OA to determine the true predictive accuracy that one may expect in a clinical setting.

Perhaps equally as important as statistical validation of the model is a clinical interpretation of the model. The first discriminant function was able to accurately discriminate High-Responders from Low-Responders and Non-Responders, and it did so primarily using two clinically interpretable predictor variables. First, individuals who responded best to the treatment intervention reported lower ADL scores at baseline. This finding is similar to that of Wright et al. [9] who also found patient-reported outcome measures (e.g., ≥ 6/10 pain) could be used as a predictor of treatment success in hip OA patients. Additionally, High-Responders displayed different hip frontal plane kinematics during loading response (gait PC 51) of walking gait as compared to other subgroups. Fig 6(A) illustrates that High-Responders underwent an increasing level of hip adduction during the loading response, while Low-Responders and Non-Responders were better able to maintain their frontal plane hip angle throughout this phase of gait. This finding is supported by previous investigations that have reported that control of the hip in the frontal plane plays a key role in the loading of the knee joint [52]. It can be postulated that those individuals with impaired control at the hip, secondary to reduced muscle strength, are more likely to respond favourably to an exercise intervention focused on hip muscle strengthening. The current findings therefore suggest that lower patient-reported ADL and hip frontal plane kinematics may be the most effective measures to determine a subset of individuals who will respond best to a hip strengthening exercise intervention.

In discriminating Non-Responders from Low-Responders, the second discriminant function utilized interactions between hip, knee, and ankle kinematics, but the most important gait PCs were associated with ankle kinematics. Gait PC 46 was a measure of the relationship between hip and ankle kinematics at toe-off, which showed Non-Responders exhibited less plantarflexion during toe-off than Low-Responders. Reduced plantarflexion at toe-off in older adults has been associated with a shift in power generation from distal joints (e.g., ankle) to more proximal joints (e.g., hip) [53], potentially due to reduced muscle function at the extremities. Therefore, the atypical gait mechanics exhibited by the Non-Responders suggests that this group may benefit from a more personalized rehabilitation program or one focused more on restoring atypical distal joint biomechanics, as opposed to the hip-protocol employed in the current study.

The second most important variable, gait PC 90, in the second discriminant function was primarily loaded on ankle frontal plane kinematics during stance. While interpreting higher-order PCs such as this can be difficult, they have been suggested to represent subtle patterns of movement that can contain valuable information for comparing between individuals [54]. Munderman et al. [52] also found gait compensations at both the hip and ankle joint in the frontal plane of patients with knee OA, but concluded changes in gait were more likely related to the hip rather than the ankle. The current findings support the importance of ankle biomechanics, particularly for Non-Responders, but similar to the conclusions of other authors [24,25], we suggest that there may be multiple patient subgroups who display different compensation strategies and react differently to treatments. Again, it further begs the question, would the Non-Responders from this hip strengthening exercise intervention become High-Responders to an ankle strength and balance exercise intervention? While the answer to that question remains outside the scope of this study, it identifies the need to conduct further research with different exercise interventions and treatment avenues to better understand how we can find the right treatment for the right patient.

Patient-reported outcome measures and gait data post-intervention and at the 3–4 year follow-up projected into the classification subspace showed that the model remained relatively stable over time. While High-Responders moved towards controls in the classification subspace following the intervention, their group centroid remained in the right third of the classification subspace (Fig 7). Therefore, this finding not only demonstrates the stability of the model, but also suggests that most of these patients would benefit from continuing with this exercise intervention. Alternatively, Non-Responders moved further from controls yet remained in their initial third of the classification space, providing evidence that this intervention is most likely still inappropriate for this subgroup. Finally, the stability of the model was also supported in the subset of patients with knee OA who completed the 3–4 year follow-up. Again, while group centroids shifted, each subgroup remained in their initial third of the subspace. Nevertheless, these results must be interpreted with caution considering that changes in treatment, activity, or severity were not measured for this subset of 3–4 year follow-up data and therefore any movement in the classification subspace are difficult to clinically interpret.

Given the findings of the current study and the growing literature demonstrating the impact of gait in OA [12–20], it is becoming increasingly apparent that evaluating patterns of movement should be a key concern for clinicians as part of their patient assessment. Unfortunately, many clinicians do not have access to 3D gait analysis systems, such as the VICON system utilized in the current study. With this in mind, there have been many simple, low cost and often portable motion analysis techniques developed and validated in the literature that can make gait analysis possible in a clinical setting [55,56]. Additionally, with the advent of wearable technology there are even more options available to a clinician when it comes to evaluating a patient’s gait [57,58]. Nevertheless, these clinically assessable technologies require a certain amount technical expertise and produce large amounts of data that can be difficult for clinicians to interpret. Therefore, developing methods and prediction models that can help package this information in a clinically interpretable manner is paramount for the integration of gait analysis in clinical practice. Although further validation is required, the current study and specifically Fig 4, is one such example that can quickly and easily provide a clinician information on the likelihood of a patient responding based on their baseline data projecting a position on a 2D plot. Therefore, it is our hope that the findings presented in this study can be a first step towards developing tools that can use gait analysis to inform decision-making in the clinical setting.

Although the current study can provide many new insights into predicting the response of patients with knee OA to an exercise intervention, it remains a preliminary investigation with known limitations. First, since the current study remains in the developmental phase of clinical prediction rules examining a single intervention, the identified predictors may be prognostic factors that are not specific to this treatment. In other words, although the predictors were clinically interpretable as being associated with a hip strengthening program, given the current design we cannot rule out that these factors may simply relate to High-Responders in general and as such these individuals would respond well to other treatment types (e.g., quadriceps strengthening, patient education). Second, there are a relatively low number of subjects involved in the 6-week investigation and as such, there is risk of the model overfitting the data. However, the use of an additional 59 patients with knee OA for data reduction and given the high redundancy in data such as kinematic waveforms [59] combined with the normal distribution and lack of outliers in the predictor variables, we are confident this risk was minimized [47]. Most importantly, the use of a cross-validation technique to select predictors and determine classification error was utilised in the present study to further minimize any overfitting errors. Nevertheless, it should be noted that with any classification model, regardless of the number of subjects, additional testing on a new test set of subjects is required to determine its true predictive capability. Third, individual Kellgren-Lawrence grading was not provided for patients with knee OA, thereby preventing an objective comparison of severity between responder subgroups. The individual scoring of radiographs was not done for this analysis since all subjects had a confirmed diagnosis of mild-to-moderate knee OA [26] and there is limited evidence for the sensitivity of radiographic grading in such a population [60,61]. Finally, both males and females were included in the same classification model. Although sex differences may exist in the gait biomechanics of patients with knee OA [62,63], no gait PCs used in the classification model were significantly correlated with sex. Therefore any differences in gait biomechanics that may have existed due to sex had a minimal effect on the classification subspace.

## Conclusion

These findings of the current study are the first to support the use of pre-intervention patient-reported outcome measures and biomechanical parameters for predicting response to an exercise intervention in a knee OA population. The best classification model of High-Responders, Low-Responders, and Non-Responders to a 6-week hip strengthening exercise intervention included a combination of baseline patient-reported ADL and biomechanical gait data. Lower patient-reported ADL and hip frontal plane kinematics were most important in classifying High-Responders from other sub-groups, while a combination of hip, knee, and ankle kinematics were used to classify Non-Responders from Low-Responders. Although these predictors identified responders to a 6-week hip strengthening program, their relationship with other types of interventions remains unclear and further validation is required. Nevertheless, this research remains a significant first step in developing an objective system to help clinicians make evidence-based decisions regarding optimal treatment for patients with knee OA.

## Supporting Information

S1 File Hip strengthening exercise protocol and daily training log given to patients for weeks 1 to 6 of intervention. (PDF)
