# Supplementary material for: Gait Biomechanics and Patient-Reported Function as Predictors of Response to a Hip Strengthening Exercise Intervention in Patients with Knee Osteoarthritis
Source: PLoS One. 2015 Oct 7;10(10):e0139923. doi: 10.1371/journal.pone.0139923 (PMC4596804; doi:10.1371/journal.pone.0139923)
Supplement: S1 File — (PDF) [file pone.0139923.s001.pdf]

OA Rehab Protocol Week 1

<table>
<tr><td>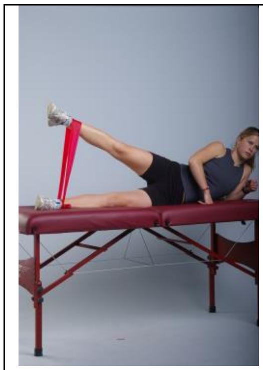</td><td>Hip Abductor Strengthening - Sidelying II

Lying on your side, with theraband between ankles (or knees for less resistance).

Move involved leg upwards, keeping knee straight.

2 seconds out and 2 seconds in, control the motion throughout.

3 sets :10 reps</td></tr>
</table>

<table>
<tr><td>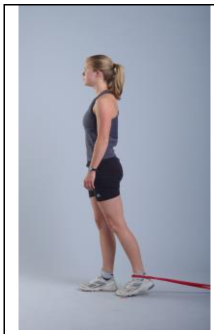 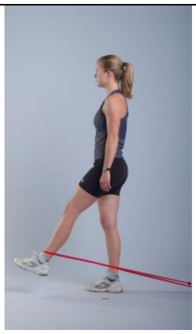</td><td>Hip Flexor Strengthening - Standing

Place opposite foot beside band

Move involved leg forward, keeping knee straight or with slight “soft knee”.

2 seconds out and 2 seconds in, control the motion throughout.

3 sets: 10 reps</td></tr>
</table>

<table>
<tr><td>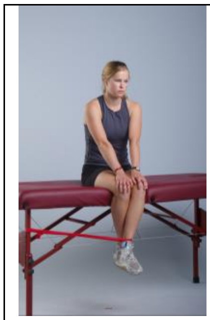 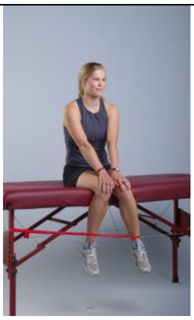</td><td>Hip External Rotator Strengthening - Seated

In seated position, move leg outwards and return to starting position slowly.

Keep knees together.

2 seconds out and 2 seconds in, control the motion throughout.

3 sets; 10 reps</td></tr>
</table>

Sunday	Monday	Tuesday	Wednesday	Thursday	Friday	Saturday
☐	☐	☐	☐	☐	☐	☐

OA Rehab Protocol Week 2

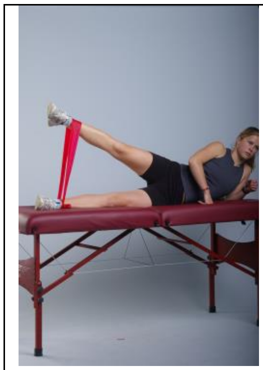

Hip Abductor Strengthening - Sidelying II

Lying on your side, with theraband between ankles (or knees for less resistance).

Move involved leg upwards, keeping knee straight.

2 seconds out and 2 seconds in, control the motion throughout.

3 sets: 15 reps

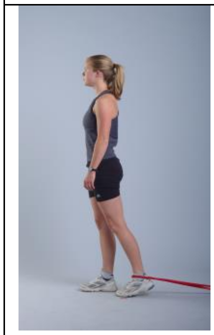
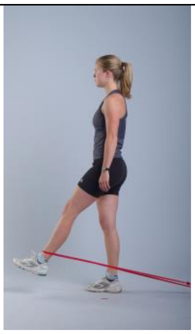

Hip Flexor Strengthening - Standing

Place opposite foot beside band

Move involved leg forward, keeping knee straight or with slight “soft knee”.

2 seconds out and 2 seconds in, control the motion throughout.

3 sets: 15 reps

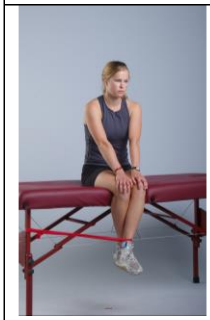
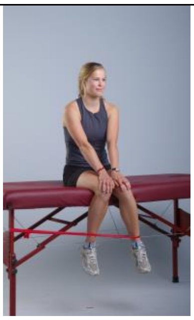

Hip External Rotator Strengthening - Seated

In seated position, move leg outwards and return to starting position slowly.

Keep knees together.

2 seconds out and 2 seconds in, control the motion throughout.

3 sets; 15 reps

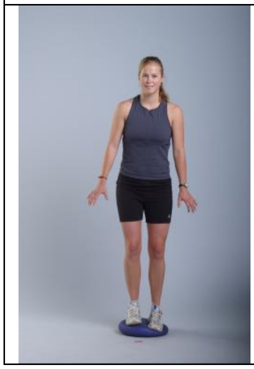

Core Stability – Two leg balancing

Standing on Airex pad with both feet.

Perform balancing for 30 second intervals. Repeat 3 times.

Use light touch on a wall initially to help balance.

3 sets: 30 sec

Sunday	Monday	Tuesday	Wednesday	Thursday	Friday	Saturday
☐	☐	☐	☐	☐	☐	☐

OA Rehab Protocol Week 3

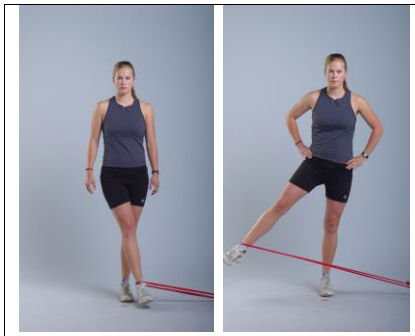

Hip Abductor Strengthening - Standing

Place opposite foot behind band

Move involved leg outward, keeping knee straight.

2 seconds out and 2 seconds in, control the motion throughout.

3 sets: __ reps ________ band

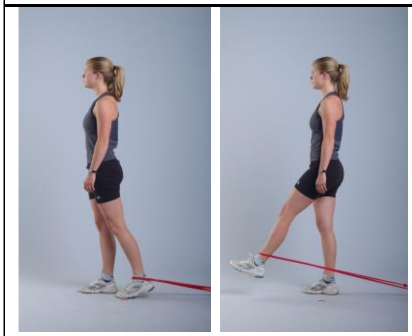

Hip Flexor Strengthening - Standing

Place opposite foot beside band

Move involved leg forward, keeping knee straight or with slight “soft knee”.

2 seconds out and 2 seconds in, control the motion throughout.

3 sets: __ reps _________ band

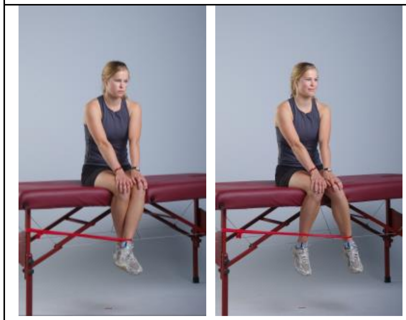

Hip External Rotator Strengthening - Seated

In seated position, move leg outwards and return to starting position slowly.

Keep knees together.

2 seconds out and 2 seconds in, control the motion throughout.

3 sets: __ reps ________band

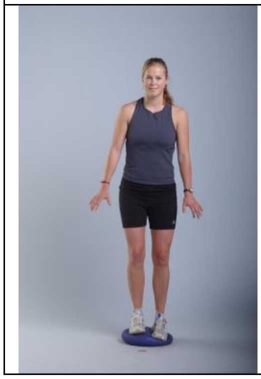

Core Stability – Two leg balancing

Standing on Airex pad with both feet.

Perform balancing for 45 second intervals. Repeat 3 times.

Use light touch on a wall initially to help balance.

3 sets: 45 sec

Sunday	Monday	Tuesday	Wednesday	Thursday	Friday	Saturday
☐	☐	☐	☐	☐	☐	☐

OA Rehab Protocol Week 4-6

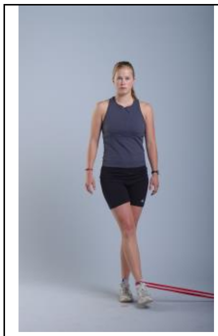 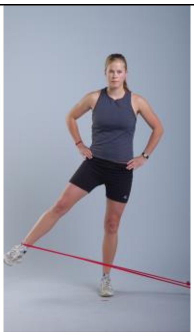	**Hip Abductor Strengthening - Standing**<br><br>Place opposite foot behind band<br><br>Move involved leg outward, keeping knee straight.<br><br>2 seconds out and 2 seconds in, control the motion throughout.<br><br>3 sets: __ reps _______band
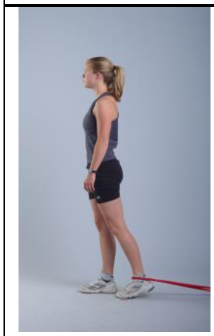 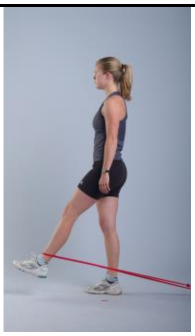	**Hip Flexor Strengthening - Standing (band above knee)**<br><br>Place opposite foot beside band<br><br>Move involved leg forward, keeping knee straight or with slight "soft knee".<br><br>2 seconds out and 2 seconds in, control the motion throughout.<br><br>3 sets: __ reps _______band
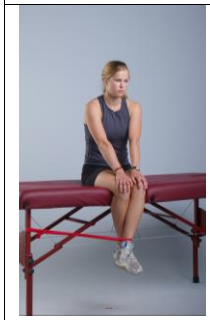 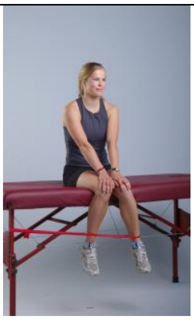	**Hip External Rotator Strengthening - Seated**<br><br>In seated position, move leg outwards and return to starting position slowly.<br><br>Keep knees together.<br><br>2 seconds out and 2 seconds in, control the motion throughout.<br><br>3 sets: __ reps _______band
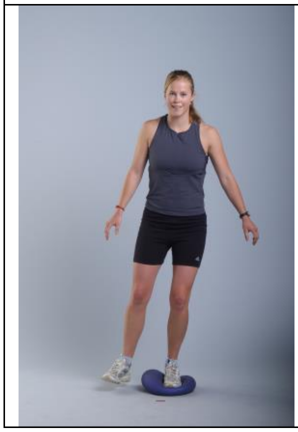	**Core Stability – One leg balancing**<br><br>Standing on Airex pad with one foot.<br><br>Perform balancing for 30 second intervals. Repeat 3 times. Repeat for other foot.<br><br>Use light touch on a wall initially to help balance.<br><br>Perform with eyes closed for more difficulty.<br><br>Week 5: 45 sec, Week 6: 60 sec

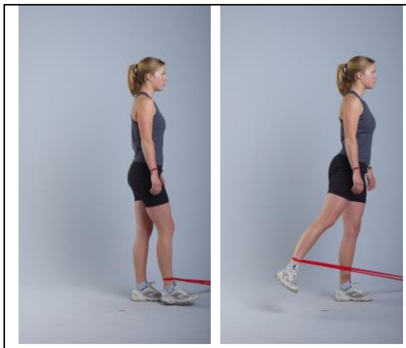

Hip Gluteus Medius Strengthening - Standing

Place opposite in front of band

Move involved leg back to 45 degree angle, keeping knee straight.

2 seconds out and 2 seconds in, control the motion throughout.

Week 4 3 sets: __ reps _______ band
Week 5 3 sets: __ reps _______ band
Week 6 3 sets: __ reps _______ band

Week 4

Sunday	Monday	Tuesday	Wednesday	Thursday	Friday	Saturday
☐	☐	☐	☐	☐	☐	☐

Week 5

Sunday	Monday	Tuesday	Wednesday	Thursday	Friday	Saturday
☐	☐	☐	☐	☐	☐	☐

Week 6

Sunday	Monday	Tuesday	Wednesday	Thursday	Friday	Saturday
☐	☐	☐	☐	☐	☐	☐
